# Supplementary material for: Empirical methods for controlling false positives and estimating confidence in ChIP-Seq peaks
Source: BMC Bioinformatics. 2008 Dec 5;9:523. doi: 10.1186/1471-2105-9-523 (PMC2628906; doi:10.1186/1471-2105-9-523)
Supplement: Additional File 1 — A variety of html documents from the USeq web site detailing the available applications, their best usage, output file type descriptions, command line menus, etc. [file 1471-2105-9-523-S1.zip › USeqUserGuides/JavaDoc.zip!/JavaDoc/trans/qc/CelFileStats.html]

CelFileStats


|  |  |  |
| --- | --- | --- |
| **Overview** \| **Package** \| **Class** \| **Use** \| **Tree** \| **Deprecated** \| **Index** \| **Help** | |  |
| **PREV CLASS**   **NEXT CLASS** | **FRAMES**    **NO FRAMES**     **All Classes** |  |
| SUMMARY: NESTED \| FIELD \| CONSTR \| METHOD | DETAIL: FIELD \| CONSTR \| METHOD |  |


---


## trans.qc Class CelFileStats

```
java.lang.Object
  trans.qc.CelFileStats
```

---

``` public class CelFileStats extends java.lang.Object ```

Container for cel file and it's associated statistics.

---

**Constructor Summary**

`CelFileStats(java.io.File serializedCelFile,
java.lang.String groupName)`
  
          Loads virtualCel float[][] and calculates a rawMedian.


| **Method Summary** | |
| --- | --- |
| `void` | `appendNotes(java.lang.String comment)` |
| `void` | `calculateControlStats(int[][][] controls, double noSynthMultiplier, double dimMultiplierHigh, double dimMultiplierLow, double brightMultiplier)`             Calculates the median value for each of the different classes of controls. |
| `void` | `calculateStats()`             Reloads intensites and calculates mean, coef var, percentiles. |
| `double` | `getCoefficientOfVariation()` |
| `java.lang.String` | `getGroupName()` |
| `float[]` | `getIntensities()` |
| `double` | `getMean()` |
| `double` | `getMedianBrightControls()` |
| `double` | `getMedianDimControls()` |
| `double` | `getMedianNoSynthControls()` |
| `double` | `getMedianScalar()` |
| `java.lang.StringBuffer` | `getNotes()` |
| `int` | `getNumBrightOutliers()` |
| `int` | `getNumDimOutliers()` |
| `int` | `getNumNoSynthOutliers()` |
| `double[]` | `getQuartiles()` |
| `double` | `getRawMedian()` |
| `java.io.File` | `getSerializedCelFile()` |
| `float[][]` | `getVirtualCel()` |
| `boolean` | `isOk()` |
| `void` | `loadFloatArrays(boolean makeCollapsedFloatArray)`             Loads the serialized virtualCel (float[][]) from disk and then collapses it to generate the intensities (float[]). |
| `void` | `nullIntensityArrays()`             Nulls the virtualCel and collapsed intensities array. |
| `void` | `setMedianScalar(double medianScalar)` |
| `void` | `setNotes(java.lang.StringBuffer notes)` |
| `void` | `setNumBrightOutliers(int numBrightOutliers)` |
| `void` | `setNumDimOutliers(int numDimOutliers)` |
| `void` | `setNumNoSynthOutliers(int numNoSynthOutliers)` |
| `void` | `setOk(boolean ok)` |
| `void` | `setRawMedian(double rawMedian)` |
| `java.lang.String` | `toString()` |

**Methods inherited from class java.lang.Object**

`equals, getClass, hashCode, notify, notifyAll, wait, wait, wait`

**Constructor Detail**

### CelFileStats

```
public CelFileStats(java.io.File serializedCelFile,
                    java.lang.String groupName)
```

:   Loads virtualCel float[][] and calculates a rawMedian.


**Method Detail**

### calculateStats

```
public void calculateStats()
```

:   Reloads intensites and calculates mean, coef var, percentiles.

---


### calculateControlStats

```
public void calculateControlStats(int[][][] controls,
                                  double noSynthMultiplier,
                                  double dimMultiplierHigh,
                                  double dimMultiplierLow,
                                  double brightMultiplier)
```

:   Calculates the median value for each of the different classes of controls.
    NoSynt, Dim, Bright, using the virtualCel and the supplied xy coordinates.
    Also counts the number of feature outliers based on the number of standard deviations

---


### nullIntensityArrays

```
public void nullIntensityArrays()
```

:   Nulls the virtualCel and collapsed intensities array.

---


### loadFloatArrays

```
public void loadFloatArrays(boolean makeCollapsedFloatArray)
```

:   Loads the serialized virtualCel (float[][]) from disk and then
    collapses it to generate the intensities (float[]).

---


### toString

```
public java.lang.String toString()
```

:   **Overrides:**: `toString` in class `java.lang.Object`

---


### getCoefficientOfVariation

```
public double getCoefficientOfVariation()
```

---


### getIntensities

```
public float[] getIntensities()
```

---


### getMean

```
public double getMean()
```

---


### getQuartiles

```
public double[] getQuartiles()
```

---


### getGroupName

```
public java.lang.String getGroupName()
```

---


### getSerializedCelFile

```
public java.io.File getSerializedCelFile()
```

---


### isOk

```
public boolean isOk()
```

---


### setOk

```
public void setOk(boolean ok)
```

---


### getNotes

```
public java.lang.StringBuffer getNotes()
```

---


### setNotes

```
public void setNotes(java.lang.StringBuffer notes)
```

---


### appendNotes

```
public void appendNotes(java.lang.String comment)
```

---


### getVirtualCel

```
public float[][] getVirtualCel()
```

---


### getMedianBrightControls

```
public double getMedianBrightControls()
```

---


### getMedianDimControls

```
public double getMedianDimControls()
```

---


### getMedianNoSynthControls

```
public double getMedianNoSynthControls()
```

---


### getNumBrightOutliers

```
public int getNumBrightOutliers()
```

---


### setNumBrightOutliers

```
public void setNumBrightOutliers(int numBrightOutliers)
```

---


### getNumDimOutliers

```
public int getNumDimOutliers()
```

---


### setNumDimOutliers

```
public void setNumDimOutliers(int numDimOutliers)
```

---


### getNumNoSynthOutliers

```
public int getNumNoSynthOutliers()
```

---


### setNumNoSynthOutliers

```
public void setNumNoSynthOutliers(int numNoSynthOutliers)
```

---


### getMedianScalar

```
public double getMedianScalar()
```

---


### setMedianScalar

```
public void setMedianScalar(double medianScalar)
```

---


### getRawMedian

```
public double getRawMedian()
```

---


### setRawMedian

```
public void setRawMedian(double rawMedian)
```


---


|  |  |  |
| --- | --- | --- |
| **Overview** \| **Package** \| **Class** \| **Use** \| **Tree** \| **Deprecated** \| **Index** \| **Help** | |  |
| **PREV CLASS**   **NEXT CLASS** | **FRAMES**    **NO FRAMES**     **All Classes** |  |
| SUMMARY: NESTED \| FIELD \| CONSTR \| METHOD | DETAIL: FIELD \| CONSTR \| METHOD |  |


---
